# Association between Polymorphisms of *OCT1* and Metabolic Response to Metformin in Women with Polycystic Ovary Syndrome

**DOI:** 10.3390/ijms20071720

**Published:** 2019-04-07

**Authors:** Hui Hua Chang, Yuan-Shuo Hsueh, Yung Wen Cheng, Huang-Tz Ou, Meng-Hsing Wu

**Affiliations:** 1 Institute of Clinical Pharmacy and Pharmaceutical Sciences, College of Medicine, National Cheng Kung University, Tainan 701, Taiwan; af31216@gmail.com (Y.W.C.); huangtz@mail.ncku.edu.tw (H.-T.O.); 2 School of Pharmacy, College of Medicine, National Cheng Kung University, Tainan 701, Taiwan; 3 Department of Pharmacy, National Cheng Kung University Hospital, Dou-Liou Branch, Yunlin 640, Taiwan; 4 International Center for Wound Repair and Regeneration, National Cheng Kung University, Tainan 701, Taiwan; shinebt88@gmail.com; 5 Department of Pharmacy, National Cheng Kung University Hospital, Tainan 704, Taiwan; 6 Department of Obstetrics and Gynecology, College of Medicine and Hospital, National Cheng Kung University, Tainan 704, Taiwan; mhwu68@mail.ncku.edu.tw

**Keywords:** polycystic ovary syndrome, metformin, polymorphisms, OCT1

## Abstract

Insulin-sensitizer treatment with metformin is widely used in polycystic ovary syndrome (PCOS). However, the treatment effectiveness shows individual differences in PCOS patients. Organic cation transporter (OCT) 1 and 2 have been reported to mediate metformin transport in the liver and kidney, respectively. In this study, we investigated the association between the polymorphisms of *OCT1* and *OCT2* and the treatment effectiveness of metformin in PCOS patients. The single nucleotide polymorphisms (SNPs) of *OCT1* (rs683369 and rs628031) and *OCT2* (rs316019) were analyzed in 87 PCOS and 113 control women. Oral glucose tolerance tests (OGTTs), which represented metformin treatment response, were conducted at the start of treatment and after six-month treatment. The results demonstrated that the SNP frequencies of *OCT1* and *OCT2* were not associated with PCOS pathophysiology, and that the polymorphisms of *OCT1* and *OCT2* were not associated with the OGTT parameters at baseline. However, PCOS patients with the G allele of *OCT1* rs683369 and/or with the A allele of *OCT1* rs628031 had increased insulin sensitivity compared to those with wild-type genotype after receiving metformin treatment. Moreover, the interactions of metformin*SNP were significant in both *OCT1* rs683369 (*p* < 0.001) and rs628031 (*p* = 0.001) during the treatment period. Taken together, genetic polymorphisms of *OCT1* contributed to different metformin treatment responses, and further study is needed to establish personalized treatment programs using a pharmacogenomic algorithm approach in PCOS patients.

## 1. Introduction

Polycystic ovary syndrome (PCOS) is a complex endocrine condition characterized by oligo/anovulation, high androgen levels, and polycystic ovaries in about 10% of females. PCOS not only affects reproduction, but can also contribute to long-term metabolism disturbance, such as diabetes and heart disease [1]. High androgen levels and insulin resistance result in metabolic and hormonal dysfunctions in PCOS patients [2], which lead to high comorbidity rates with diabetes mellitus [3,4]. Physiologically, ovarian theca cells provide support to the growing follicle, assisting in mature oocyte generation [5]. Interestingly, ovarian theca cells from PCOS subjects are insulin-sensitive compared to peripheral tissues, which are insulin-resistant [6,7]. These theca cells in PCOS patients are hyper-responsive to the stimulatory effects of insulin and cause ovarian hyperthecosis. Insulin resistance in peripheral tissues amplifies the androgenic potential in the theca cells and further aggravates the symptoms of PCOS [8]. Additionally, the high sensitivity of theca cells to gonadal steroid gonadotropin stimulation aids hyperandrogenism in PCOS. Anovulation and menstrual cycle abnormalities are the frequent symptoms troubling women of reproductive age.

Metformin is an off-label medication used in PCOS patients as an insulin sensitizer [9]. Metformin can not only lower elevated parameters such as insulin, androgens, and circulating free T levels, but can also increase levels of sex hormone-binding globulin (SHBG) and insulin-like growth factor-binding protein (IGFBP) [10]. The well-known action of metformin is to suppress the production of hepatic glucose. Moreover, metformin has been demonstrated to increase the synthesis of SHBG and to improve menstrual frequency, ovulation, conception, and live birth rates [11,12]. These findings suggest a reasonable application of metformin for PCOS patients, and it has been shown to improve the quality of life in females with PCOS. However, the treatment effectiveness of metformin shows individual differences in PCOS patients, and 30% of these subjects did not respond to metformin [13]. However, the predictor of the outcome of metformin treatment has not been adequately clarified.

Organic cation transporter (OCT) proteins mediate the transport of organic cations across the cell membrane. Metformin has been demonstrated to be a substrate of liver-specific OCT1 and kidney-abundant OCT2 [14,15]. These studies indicate that the pharmacokinetic and pharmacodynamic profiles of metformin are mediated by the activity of OCT1 and OCT2 [16,17]. In *Oct1*-deficient mice, the hepatic metformin concentration in the liver was found to be significantly lower than that in control mice, and the glucose-lowering effects of metformin were completely abolished [18]. This indicates that OCT1 expression and activity is essential for the hepatic uptake of metformin [19,20,21]. However, the polymorphisms of *OCT1* and *OCT2* genes may affect the activity of metformin transport and further influence the treatment response of metformin in individuals.

Therefore, in this study, we investigated the association between the polymorphisms of *OCT1* and *OCT2,* and the treatment effectiveness of metformin in patients with PCOS. Oral glucose tolerance tests (OGTTs), which were used for identification in PCOS patients, were conducted to evaluate the treatment effectiveness of metformin. Our findings show the clinical relevance of *OCT1* and *OCT2* for predicting treatment outcomes in PCOS patients treated with metformin.

## 2. Results

A total of 87 patients with PCOS who had a first prescription for metformin and 113 controls were enrolled. The demographic data and baseline levels of insulin and glucose are listed in Table 1. Age was significantly different between the controls and PCOS patients. Additionally, women with PCOS had significantly higher BMIs, systolic blood pressure (SBP), and diastolic blood pressure (DBP), and frequent disturbances in glucose metabolism and insulin level, compared with the control group, even after adjustment for age.

Levels of insulin and glucose determined by 2-hr OGTT in PCOS patients before treatment (baseline) and in those treated with metformin for six months are shown in Table 2. Insulin levels were significantly reduced after metformin treatment (92.1 to 59.2 µIU/mL, *p* < 0.001). Additionally, the glucose/insulin ratio (G/I ratio) was significantly increased after metformin treatment (2.2 to 3.5, *p* = 0.001), although glucose levels did not change significantly (116 to 115 mg/dL). Our findings show clinical benefits for PCOS patients receiving metformin treatment. However, a wide standard deviation was observed in the PCOS patients, which suggests a great inter-patient variability of clinical response.

To examine the individual differences in patient response, we further investigated the effect of *OCT1* and *OCT2* polymorphisms on metformin-treated PCOS patients. The genotypes and allele frequencies of *OCT1* and *OCT2* in healthy controls and PCOS patients are shown in Table 3. None of the polymorphisms of *OCT1* and *OCT2* genes deviated from the Hardy–Weinberg equilibrium. Additionally, the frequencies of the polymorphisms of *OCT1* and *OCT2* were not significantly different between the control and PCOS patients (Table 3), which indicates that *OCT1* and *OCT2* were not associated with PCOS pathophysiology.

Furthermore, to examine the association between genetic polymorphisms and metformin treatment responses in PCOS, we analyzed the association between genotypes and the levels of OGTT parameters (Table 4). At baseline, neither the polymorphisms of *OCT1* nor those of *OCT2* were associated with the OGTT parameters, including 2-hr glucose, insulin, and 2-hr G/I ratio. However, the results demonstrated that the changes in OGTT-determined G/I ratio were significantly different in patients with polymorphisms of *OCT1* after metformin treatment (Table 4). Patients with the G allele of *OCT1* rs683369 (4.3 ± 3.8 vs. 3.3 ± 3.0) and/or the A allele of *OCT1* rs628031 (3.9 ± 2.9 vs. 3.2 ± 3.2) had higher OGTT-determined G/I ratios than those with wild-type genotype after receiving metformin treatment. Moreover, the interactions of metformin*single nucleotide polymorphism (SNP) were significant for both *OCT1* rs683369 (*p* < 0.001) and rs628031 (*p* = 0.001) (Table 4). Taken together, the results indicate that treatment response was affected by genetic polymorphisms during the metformin treatment period.

## 3. Discussion

In the current prospective clinical study, *OCT1* genetic polymorphisms were found to influence the effectiveness of metformin in PCOS patients. There were great individual differences in the PCOS patients, although some of these patients receiving metformin treatment experienced clinical benefits, as shown in previous reports [22,23]. Patients with polymorphisms of *OCT1* were found to have significantly different insulin sensitivity after metformin treatment, while the interactions of metformin*OCT1 were also found to be significant. Patients with the G allele of *OCT1* rs683369 and/or the A allele of *OCT1* rs628031 had higher OGTT-determined G/I ratios than those with wild-type genotype after receiving metformin treatment. However, the distribution of the SNP frequencies in *OCT1* and *OCT2* were not significantly different in PCOS patients compared to controls, and the genotype frequencies of these polymorphisms in the PCOS and control subjects were similar to those reported in Asian populations in the 1000 Genome Project. The genotypic distribution of *OCT1* rs683369 and rs628031 in the participants was consistent with that presented in the Ensembl project (http://asia.ensembl.org) for participants of Asian ethnicity (GC/GG/CC = 20.2/2.9/76.9% in rs683369, and AG/AA/GG = 28.8/4.8/66.3% in rs628031). The worldwide ethnic distribution of the frequency of the G allele of *OCT1* rs683369 is highly variable (about 2% in Africans, about 10% in Mixed American, and 15% in Asians), while the distribution of the A allele of *OCT1* rs628031 is similar (about 25% across ethnicities). Additionally, the genotypic distribution of *OCT2* rs316019 is consistent with the Asian group (AC/AA/CC = 19.5/0/80.5%) and is similar to the African Ancestry population according to the Ensembl project. Furthermore, previous evidence, as well as that provided by our study, indicate that genetic background, including *OCT1* and *OCT2*, is not associated with PCOS itself [24]. Taken together, the results indicate that treatment response was affected by *OCT1* genetic polymorphisms during the metformin treatment period, and that *OCT1* and *OCT2* were not associated with PCOS pathophysiology.

Although the effectiveness of metformin response is important for PCOS patients, 30% of PCOS patients have a poor response to metformin. Therefore, it is urgently required to develop and establish clinical predictors for treatment outcome. OCTs are involved in transporting a broad spectrum of endogenous compounds, such as monoamine neurotransmitters, choline, coenzymes, and xenobiotics [25,26]. Polymorphisms of *OCT1* and *OCT2* have been found to be correlated with diseases such as diabetes mellitus, diabetic nephropathy, primary biliary cirrhosis, and hypertension [27,28,29]. In order to identify whether polymorphisms of *OCT1* and *OCT2* contribute to the variability in PCOS itself and treatment response, a prospective study was conducted in which 150 PCOS patients of European descent were treated with metformin [24]. The results revealed that the *OCT1* genotype was a determinant of the insulin response to metformin in PCOS patients with no variants or one variant, although there was lack of the appropriate value for the area under the curve (AUC) of glucose in the OGTT at baseline and at the end of the treatment. This indicates that polymorphisms in *OCT1* are associated with the variability in the response to metformin in PCOS patients. Other studies have also suggested that *OCT* variants are significantly associated with elevated baseline and glucose-induced C-peptide levels in PCOS subjects after metformin treatment [13,30]. The polymorphism of *OCT1* 420del reduced the transport activity of metformin [31]. Another genetic variant in *OCT2* 808G/T also resulted in an amino acid change, A270S, on the renal elimination of metformin [32], and the plasma concentrations of metformin in subjects with heterozygous 808G/T or homozygous 808T/T genotypes were higher than those with the GG genotype. Similarly, in the current study, patients with the G allele of *OCT1* rs683369 and/or with the A allele of *OCT1* rs628031 had higher OGTT-determined G/I ratios than those with wild-type genotype after receiving metformin treatment. To summarize, polymorphisms of *OCT1* and *OCT2* have been identified which could influence the hepatic intake or renal clearance of metformin in vivo and in vitro [18,27,30,31,33,34,35,36]. These findings demonstrate the role of *OCT1* and *OCT2* polymorphisms in modulating responses to metformin treatment in PCOS patients.

In addition to OCT1 and OCT2, the absorption, distribution, and excretion of metformin is also mediated by OCT3, multidrug and toxin extrusion proteins 1–2 (MATE1-2), and plasma membrane monoamine transporter (PMAT) [37]. OCT3 is highly expressed on the plasma membranes of skeletal muscle and liver, which are target tissues for metformin action [21]. Notably, the genetic variants of *OCT3* T44M, T400I, and V423F may modulate metformin uptake. MATE1 is responsible for the excretion of metformin into bile and urine [38]. The polymorphisms of *MATE1* have been found to be associated with an amplified glucose-lowering effect of metformin in diabetic patients [39]. A MATE2 splice variant, MATE2-K, has also been shown to be a transporter of metformin [40]. Moreover, promoter polymorphisms of *MATE1* (rs2252281) and *MATE2* (rs12943590) have been shown to be important determinants of metformin disposition and response in healthy controls and diabetic patients [41,42,43]. Further studies integrated with genetic polymorphisms of *OCT3*, *MATE1*, *MATE 2*, and *PMAT* should investigate their potential as predictors of the effectiveness of metformin treatment in PCOS patients. Such studies will be helpful to establish personalized treatment programs using a pharmacogenomic algorithm approach.

Although the data were presented carefully, this study has certain limitations. The first limitation is the relatively small sample size of PCOS patients, and the fact that they were from a single site. Although the relatively small sample size may limit the interpretation of the results, the association between polymorphisms of *OCT1* and OGTT parameters had a statistical power of 0.8. The second limitation is the lack of control for some confounding factors such as diet, exercise, and general health status. The third limitation is that we evaluated metformin treatment response by OGTT-derived insulin sensitivity. Limited to clinical practice, the fourth limitation is that, although we measured 2-hr glucose and insulin by OGTT in PCOS patients before and after metformin treatment, the fasting levels of insulin and glucose were collected only before treatment. Thus, we could not calculate the area under the curve for insulin and glucose, nor could we calculate the homeostasis model assessment-estimated insulin resistance (HOMA-IR) in PCOS patients with metformin treatment. The glycemic response should be validated to further clarify the contribution of the insulin homeostasis of metformin in PCOS subjects. Further study is also needed to clarify whether the polymorphisms of *OCT1* could be useful clinical indicators for monitoring metformin treatment response in patients with PCOS.

## 4. Materials and Methods

### 4.1. Subjects

The research protocol was approved by the Institutional Review Board for the Protection of Human Subjects at National Cheng Kung University Hospital, Taiwan. All participants signed written informed consent forms. A total of 200 genetically unrelated women aged between 18 and 45 years, including 87 PCOS patients and 113 control subjects, were recruited from the Obstetrics and Gynecology clinics of the National Cheng Kung University Hospital. The controls were enrolled from an infertility clinic prior to entering an in vitro fertilization program due to tubal and/or male factors without menstrual cycle irregularities, clinical or biochemical hyperandrogenism, polycystic ovaries on ultrasound examination, or a history of systemic/endocrine disease.

Patients were diagnosed with PCOS during the study period according to the Rotterdam criteria, in which PCOS was defined by the presence of at least two out of the following three criteria: (1) oligo-anovulation (a cycle length >35 days or amenorrhoea); (2) clinical hyperandrogenism (hirsutism recorded as m-FG score ≥ 6 with/without acne or androgenic alopecia) and/or biochemical hyperandrogenism (total testosterone level of more than 0.95 ng/mL); and (3) polycystic ovaries (≥12 follicles measuring 2–9 mm in diameter, or ovarian volume >10 mL in at least one ovary). We further excluded those (1) diagnosed with similar clinical presentations (e.g., congenital adrenal hyperplasia); (2) diagnosed with diabetes, or with fasting plasma glucose ≥126 mg/dL or 2-hr glucose determined by OGTT ≥200 mg/dL before PCOS diagnosis; and (3) taking any medications that may influence insulin level or contraceptive pills three months prior to PCOS diagnosis.

All PCOS patients were treated with metformin (500 mg, three times a day) once PCOS had been diagnosed. Patients could be switched to extended-release metformin if they could not tolerate immediate-release formulation (due to gastrointestinal intolerance side effects).

Each subject underwent an anthropometric evaluation at baseline and after the six months of the metformin treatment. The body weight and body height of each subject were measured, and BMI (kg/m^2^) was calculated accordingly.

### 4.2. Insulin Sensitivity Analysis

A modified OGTT that used a 75 g glucose load and measured glucose and insulin at a 2-hr interval has been used in clinical studies. A 2-hr G/I ratio obtained by OGTT has also been reported to give a reasonably accurate insulin sensitivity. An abnormal ratio of glucose to insulin was defined as a G/I ratio ≤1.0 at 2-hr in a PCOS population [44]. Blood samples were collected, and then a 75 g OGTT was performed with blood samples taken after 2-hr for the determination of glucose and insulin levels. The level of plasma glucose was determined using the glucose oxidase method (Synchron CX3, Beckman, CA, USA). The serum insulin concentration was measured using a solid-phase radioimmunoassay method (Diagnostic Products Corporation, Los Angeles, CA, USA).

### 4.3. Genotyping

Genomic DNA was extracted from each blood sample using a QIAamp DNA blood kit (QIAGEN, Hilden, Germany) according to the manufacturer’s instructions. The DNA was stored at −80 °C until use. The SNPs of *OCT1* (P160L, rs683369; M408V, rs628031) and *OCT2* (A270S, rs316019) were genotyped using commercially-available TaqMan^®^ SNP Genotyping Assays (Applied Biosystems, Foster City, CA, USA). Amplification and dissociation were carried out using the ABI 7900HT Fast Real-Time PCR System (Applied Biosystems). The PCR system automatically calculated the negative derivative of the change in fluorescence. The SNP genotype of each tested sample was determined using the software program StepOne and was confirmed manually. In case of disagreement, the analysis would be repeated.

### 4.4. Statistical Analysis

Data was shown as mean ± SD and frequency. T-test and chi-square analyses were used to compare demographic and clinical characteristics between groups. The OGTT parameters were evaluated by repeated measure two-way ANOVA. We examined whether the distribution of genotypes was consistent with the Hardy–Weinberg equilibrium using the chi-square goodness-of-fit test. Statistical analyses were performed using the Statistical Package for Social Sciences 16.0 for Windows software package (SPSS Inc., Chicago, IL, USA). Two-tailed *p* values <0.05 were considered statistically significant.

## 5. Conclusions

In summary, our study indicates that the treatment response of metformin was significantly different in patients with PCOS. Although *OCT1* and *OCT2* were not associated with PCOS pathophysiology, patients with the G allele of *OCT1* rs683369 and/or with the A allele of *OCT1* rs628031 had increased insulin sensitivity compared to those with wild-type genotype after receiving metformin treatment. Moreover, the interactions of metformin*SNP were significant in *OCT1*. Additionally, our data suggest that a 2-hr G/I ratio obtained by OGTT could be a reasonable method to assess insulin sensitivity in PCOS patients. Taken together, OCT1 could be important for metformin therapeutic action, and genetic polymorphisms of *OCT1* might contribute to differences in the effectiveness of metformin treatment in PCOS patients. Further study is needed to establish personalized treatment programs for PCOS patients using a pharmacogenomic algorithm approach.

## Figures and Tables

**Table 1:** Baseline characteristics of the controls and polycystic ovary syndrome (PCOS) patients.

Mean ± SD	Controls (*n* = 113)	PCOS Patients (*n* = 87)	*p* Value	Adjusted *p* Value ^1^
**Age (years)**	30 ± 5.9	27.9 ± 5.4	0.009 *	
**Height (cm)**	160.2 ± 5.2	158.7 ± 6	0.063	
**Weight (kg)**	57.4 ± 9.8	67.9 ± 17.9	<0.001 *	<0.001 *
**BMI (kg/m^2^)**	22.5 ± 4.1	26.9 ± 6.6	<0.001 *	<0.001 *
**SBP (mmHg** **)**	112.9 ± 12.3	122.7 ± 15.4	<0.001 *	<0.001 *
**DBP (mmHg** **)**	67.3 ± 10.7	76.3 ± 13.9	<0.001 *	<0.001 *
**Insulin (µIU/mL)**	27 ± 21.7	92.1 ± 67	<0.001 *	<0.001 *
**Glucose (mg/dL)**	94.8 ± 24.9	116 ± 30.8	<0.001 *	<0.001 *

^1^ Adjusted *p* value: adjusted by age. * *p* < 0.05. Abbreviations: BMI, body mass index; SBP, systolic blood pressure; DBP, diastolic blood pressure.

**Table 2:** Oral glucose tolerance test (OGTT) parameters in all women with PCOS before and after metformin treatment.

OGTT Parameters	Before Metformin	After Metformin	*p* Value
**2-hr insulin (µIn/mL)**	92.1 ± 67.0	59.2 ± 42.7	<0.001 *
**2-hr glucose (mg/dL)**	116.0 ± 30.8	115.2 ± 35.2	0.777
**2-hr G/I ratio**	2.2 ± 2.1	3.5 ± 3.7	0.001 *

Abbreviations: G/I ratio, glucose/insulin ratio. * *p* < 0.05.

**Table 3:** Genotypes and allele frequencies of organic cation transporter (*OCT) 1* and *2* in healthy controls and PCOS patients.

Gene	rs Number	Alleles		Total	HE		HO		WT		MAF	*p* Value
	(HE/HO/WT)				*n*	(%)	*n*	(%)	*n*	(%)		
***OCT 1***	rs683369	G/C	HC	113	32	(28.3)	0	(0)	81	(71.7)	G = 0.142	0.524
	(GC/GG/CC)		PCOS	87	22	(25.3)	1	(1.1)	64	(73.6)	G = 0.138	
	rs628031	A/G	HC	113	45	(39.8)	6	(5.3)	62	(54.9)	A = 0.252	0.845
	(AG/AA/GG)		PCOS	87	36	(41.4)	6	(6.9)	45	(51.7)	A = 0.276	
***OCT 2***	rs316019	A/C	HC	113	27	(23.9)	2	(1.8)	84	(74.3)	A = 0.137	0.874
	(AC/AA/CC)		PCOS	87	23	(26.4)	1	(1.2)	63	(72.4)	A = 0.144	

Abbreviations: HC, healthy controls; MAF: minor allele frequency; HE, heterogeneous; HO, homogeneous; WT: wild type.

**Table 4:** Oral glucose tolerance test (OGTT) parameters before and after metformin treatment, subgrouped by genotypes of *OCT1* and *OCT2.*

	*OCT1* rs683369 (C/G)				*OCT1* rs628031 (G/A)				*OCT2* rs316019 (C/A)			
	CC	G Allele	*p* Values	*p* Values ^a^	GG	A Allele	*p* Values	*p* Values ^a^	CC	A Allele	*p* Values	*p* Values ^a^
OGTT parameter												
2-hr glucose (mg/dL)			0.772	0.464			0.902	0.903			0.865	0.301
Before metformin	117.8 ± 33	111.5 ± 22.1			117 ± 34.3	115.6 ± 27.6			116.5 ± 31.7	114.3 ± 28.4		
After metformin	114.2 ± 35.4	116.2 ± 29.8			115.6 ± 36.7	113.5 ± 30.1			112.6 ± 34.3	120.6 ± 32.9		
2-hr insulin (µIU/mL)			0.465	0.981			0.211	0.265			0.862	0.631
Before metformin	93.8 ± 65.8	93.4 ± 73.6			86 ± 54.2	96.5 ± 83.2			96.5 ± 67.9	84.1 ± 68.2		
After metformin	59.1 ± 42.5	51.4 ± 43.1			62.2 ± 42.7	55.9 ± 43.8			58.7 ± 43.6	60.9 ± 41.5		
2-hr G/I ratio			<0.001 *	<0.001 *			0.047 *	0.001 *			0.565	0.401
Before metformin	2.3 ± 2.4	2.1 ± 1.1			2.3 ± 2.5	2.0 ± 1.9			2.2 ± 2.3	2.2 ± 1.6		
After metformin	3.3 ± 3.0	4.3 ± 3.8			3.2 ± 3.2	3.9 ± 2.9			3.9 ± 4.3	2.7 ± 1.5		

* *p* < 0.05; **^a^** the interactions of metformin and single nucleotide polymorphism (SNP) on OGTT parameter.
